# Antimicrobial Susceptibility of Commensal *Escherichia coli* from Pig Fecal Samples and Enhanced Sensitivity for Direct Detection of the *bla*_CTX-M_ Gene by Nested PCR

**DOI:** 10.3390/ani14182630

**Published:** 2024-09-10

**Authors:** Nutchaba Suchanta, Naeem Ullah, Pitak Santanirand, Nutthee Am-In, Nuntaree Chaichanawongsaroj

**Affiliations:** 1 Center of Excellence for Innovative Diagnosis of Antimicrobial Resistance, Department of Transfusion Medicine and Clinical Microbiology, Faculty of Allied Health Sciences, Chulalongkorn University, Pathumwan, Bangkok 10330, Thailand; nutchaba.suchanta@hotmail.com (N.S.); naeem.bch@gmail.com (N.U.); 2 Program of Molecular Sciences in Medical Microbiology and Immunology, Department of Transfusion Medicine and Clinical Microbiology, Faculty of Allied Health Sciences, Chulalongkorn University, Pathumwan, Bangkok 10330, Thailand; 3 Department of Pathology, Faculty of Medicine Ramathibodi Hospital, Mahidol University, Bangkok 10400, Thailand; pitak.san@mahidol.ac.th; 4 Department of Obstetrics Gynaecology and Reproduction, Faculty of Veterinary Science, Chulalongkorn University, Pathumwan, Bangkok 10330, Thailand; nutthee.a@chula.ac.th

**Keywords:** *E. coli*, ESBLs, antimicrobial resistance, *bla*_CTX-M_ gene, nPCR, pig feces

## Abstract

**Simple Summary:**

The dissemination of antimicrobial resistance (AMR) genes in economic animals affects food safety in our life cycle. The pig gut microbiome can be a reservoir of antimicrobial-resistant bacteria. As a result of the comparison of antimicrobial susceptibility profiles and the existence of ESBL genes, we found no difference between antibiotic- and nonantibiotic-using farms. We suggest a basal level of ESBL *E. coli* persistence on pig farms, which may not depend on antibiotic usage. Direct detection of antimicrobial-resistant genes from pig fecal samples reduced cumbersome bacterial culture processes. The enhanced sensitivity of the nPCR technique facilitates the surveillance of AMR genes, leading to effective control.

**Abstract:**

The commensal *Escherichia coli* in the gut of pigs is a major reservoir of antimicrobial resistance and can result in possible transmission to humans through the food chain. Direct detection of *E. coli* from fecal samples is challenging and can be used as a bioindicator of antimicrobial resistance. This study aimed to compare the antimicrobial susceptibility profiles in commensal *E. coli* from antibiotic- and nonantibiotic-using pig farms and developed the direct detection of ESBL genes in pig fecal samples using nested PCR (nPCR) and multiplex PCR (mPCR) techniques. All direct genotypic results were validated with the results of PCR sequencing of isolated *E. coli* colonies. The ESBL-producing *E. coli* were found in 98.6% (145 isolates) and 96.6% (144 isolates) of antibiotic-using and nonantibiotic-using farms, respectively, predominantly CTX-M-55. The nPCR decreased the limit of detection (LOD) from sPCR about 100 times, and the lower LODs of 10^2^, 10^1^, and 1 CFU/mL were reached after incubating samples in an enrichment medium for 2, 4, and 8 h, respectively. The mPCR, sPCR, and nPCR techniques showed sensitivities of 30.15%, 69.85%, and 91.91%, respectively, compared to PCR sequencing. The stability and recycling of ESBL genes were independent of antibiotic usage in commensal *E. coli* originating in pig farms.

## 1. Introduction

The inappropriate and high usage of antibiotics in humans and animals has driven the world to the edge of a post-antibiotic era, in which no antibiotic can be used for infectious disease treatment. The dissemination of drug-resistant bacteria has become an important global public health crisis in the One Health system due to horizontal gene transfer in agriculture, livestock, and the environment. The extended-spectrum beta-lactamase (ESBL)-producing bacteria are a World Health Organization (WHO) critical priority concern. The unnecessary use or overuse/misuse of antibiotics in food-animal production, particularly in pigs, leads to the colonization and continuous spreading of drug resistance, ineffective and prolonged treatment, and economic loss [1]. Consequently, establishing a control point to limit the transmission of drug-resistant pathogens is a serious priority. Extended-spectrum beta-lactamase (ESBL)-producing *E. coli* are a major critical commensal antibiotic-resistance source in food animals. The broad activities of ESBL enzymes, which destroy ampicillin, cephalosporins, and aztreonam, are responsible for several genes, such as *bla*_CTX-M_, *bla*_TEM_, *bla*_OXA_, and *bla*_SHV_ [2]. The predominant ESBL gene in pigs and pork in many countries, including Thailand, was CTX-M (ranging from 91 to 98%), with different CTX-M subtypes among different areas [3]. A higher incidence of ESBL-producing *E. coli* has been reported on farms with higher antibiotic usage than those without [4]. However, the rising prevalence of ESBL-producing *Enterobacteriaceae* has been reported in animals without antibiotic usage [5]. Hence, ESBL-producing *E. coli* in fecal samples could be used as an indicator for antimicrobial resistance tracking.

Phenotypic antimicrobial susceptibility testing (AST) based on Clinical and Laboratory Standards Institute (CLSI) guidelines is a conventional routine method, while genotypic detection is beneficial for epidemiological purposes. Nowadays, both systems are still based on bacterial culture, which is a cumbersome, time-consuming process that limits the widespread adoption of AMR monitoring [6]. Direct detection from samples or clinical specimens is an ideal detection assay, as it is a “sample in and answer out” method. However, the bottleneck and troublesome process is DNA extraction, especially from fecal specimens, due to a lot of fiber and interference substances, reducing the efficiency of nucleic acid extraction and amplification [7,8]. Nested Polymerase Chain Reaction (nPCR) using two sets of primers and second rounds of PCR amplification could enhance specificity and sensitivity in several pathogenic detections such as feline leukemia virus (FeLV) [9], *Borrelia burgdorferi* [10], and malaria [11].

This study aimed to compare the antimicrobial susceptibility profiles and the presence of ESBL genes between antibiotic-using and nonantibiotic-using farms. The direct detection of *bla*_CTX-M_ in pig fecal samples using nPCR, multiplex PCR (mPCR), and singleplex PCR (sPCR) techniques were validated with the PCR sequencing results of *E. coli* isolates from the same samples. The persistence of antimicrobial resistance in the intestinal tracts of pigs highlighted the importance of effective detection and control of the spread in the food chain.

## 2. Materials and Methods

### 2.1. Study Design and Farm Selection

A cross-sectional study was conducted in pig-fattening farms (500–650 animals per farm) located in Thailand’s central and northeastern parts in 2021–2022. Five antibiotic-using (Code: PIG, A1–3, and D1) and five nonantibiotic-using (Code: S1, S2, and ND1–3) farms were randomly selected. The research proposal was approved by the Institutional Animal Care and Use Committee of Chulalongkorn University (Animal Use Protocol 2031065).

### 2.2. Collection of Fecal Samples

About 30 fecal samples were randomly collected on each farm from healthy pigs in the last week before slaughter. Two hundred and ninety-six samples were kept in Cary Blair medium and transported on ice to the laboratory for analysis within 24–48 h.

### 2.3. Bacterial Culture, Identification, and Antibiotic Susceptibility Testing

All rectal swabs were suspended in 0.5 mL of 0.85% NaCl and cultured on MacConkey agar (Oxoid, Basingstoke, UK). A cefotaxime disk (30 µg) was incubated on agar at 37 °C for 18–24 h. Colonies that grew around the disk were selected and identified using MALDI-TOF mass spectrometry (Bruker, Karlsruhe, Germany). All *E. coli* isolates were subjected to antimicrobial susceptibility testing using an automated Sensititre ARIS HiQ system (Thermo Scientific, Waltham, MA, USA). Minimum inhibitory concentrations were determined for the following antibiotics: amikacin, gentamicin, netilmicin, ampicillin, amoxicillin/clavulanic acid, ampicillin/sulbactam, piperacillin/tazobactam, cefuroxime, cefoxitin, ceftriaxone, cefotaxime, ceftazidime, cefepime, ertapenem, doripenem, imipenem, meropenem, ciprofloxacin, levofloxacin, and trimethoprim/sulfamethoxazole. The combination drug method using ceftazidime/ceftazidime–clavulanate and cefotaxime/cefotaxime–clavulanate was also added in the susceptibility panel to confirm ESBL-producing isolates. The results were interpreted according to the Clinical and Laboratory Standards Institute (CLSI) guideline [12]. *E. coli* ATCC 25922 was used as quality control. The ESBL-producing-*E. coli* isolates were preserved as 20% glycerol stocks at −80 °C until use.

### 2.4. DNA Extraction

All 289 *E. coli* isolates from 10 farms and 136 pig fecal samples from antibiotic-using farms were subjected to DNA extraction using boiling. Briefly, *E. coli* isolates or fecal specimens were suspended in 250 µL of Tris-EDTA buffer, vortexed, and heated at 98 °C for 10 min. The suspension was centrifuged at 13,000 rpm for 5 min. The DNA in the supernatant was quantified with a spectrophotometer at 260/280 nm and stored at −20 °C until use.

### 2.5. Multiplex PCR for Detecting CTX-M, TEM, OXA, and SHV Genes and Characterization of ESBL Variants

All 289 *E. coli* isolated from 10 farms were screened for the presence of the most encountered ESBL genes: *bla*_CTX-M_, *bla*_TEM_, *bla*_SHV,_ and *bla*_OXA_, using a multiplex PCR (mPCR). The *uspA* gene, universal stress protein A, was used as an internal control. The sequences of the four specific primers and the sizes of their PCR products are shown in Table 1. The multiplex PCR reactions were conducted in a final volume of 25 µL, comprising 1× Standard Taq reaction buffer, 0.2 mM dNTPs, 50 ng of DNA template, and 0.625 U of Taq polymerase (New England Biolabs, Wiltshire, UK). The primer concentrations were as follows: 0.6 µM for *bla*_CTX-M_, 0.8 μM for *bla*_OXA_, 0.12 μM for *bla*_TEM_, 0.4 μM for *bla*_SHV_, and 0.1 μM for *uspA*. The mixture of positive control genes, including *bla*_CTX-M_, *bla*_TEM_, *bla*_OXA_, and *bla*_SHV_ originating from *E. coli* EC137 and *K. pneumoniae* KP125, were kindly provided by Prof. Visanu Thamlikitkul, Faculty of Medicine Siriraj Hospital, Mahidol University, Bangkok, Thailand. The amplification steps were accomplished using a thermocycler (Bio-Rad, Hercules, CA, USA) with the following conditions: 94 °C for 5 min, followed by 30 cycles at 94 °C for 1 min, 60 °C for 1 min, and 72 °C for 1 min, with a final extension at 72 °C for 5 min. All PCR amplicons were analyzed by 2% agarose gel electrophoresis. The positive PCR amplicons of a single PCR were subjected to sequencing using specific primers to identify ESBL gene variants. The Basic Local Alignment Search Tool was used to interpret DNA sequences with the GenBank database using BLASTn (https://blast.ncbi.nlm.nih.gov/Blast.cgi) accessed on 8 September 2024. Clustal Omega (https://www.ebi.ac.uk/jdispatcher/msa/clustalo) accessed on 8 September 2024 was used for sequence alignment.

### 2.6. Direct Detection of the bla_CTX-M_ Gene by sPCR, nPCR, and mPCR from Pigs’ Fecal Samples

All 136 DNA samples, extracted directly from pig fecal samples, were subjected to sPCR, nPCR, and mPCR. The sPCR was performed in a total volume of 25 µL, consisting of 1× Standard Taq reaction buffer, 0.2 mM dNTPs, 0.3 µM of each primer, 0.625 U Taq polymerase (New England Biolabs, Wiltshire, UK), and 50 ng of DNA template. The thermal cycling conditions involved an initial denaturation at 95 °C for 15 min, followed by 30 cycles of denaturation at 95 °C for 30 s, annealing at 58 °C for 30 s, and extension at 72 °C for 2 min, with a final extension at 72 °C for 10 min. One microliter of negative results in the sPCR underwent nPCR by setting a secondary round of PCR with the same primer set at 0.075 µM. At the same time, the mPCR reaction was performed as per the previous condition for *E. coli* colonies. *E. coli* harboring the *bla*_CTX-M_ gene and sterile distilled water were incorporated as positive and negative controls, respectively.

### 2.7. Specificity of nPCR

The specificity of nPCR for the direct detection of the *bla*_CTX-M_ gene from pig feces was assessed. Ten milligrams of ESBL-negative fecal samples were suspended in 500 µL of 0.85% NaCl. Individual colonies of *E. coli* ATCC 25922, *Klebsiella pneumoniae* ATCC 700603, *Acinetobacter baumannii* ATCC 19606, *Pseudomonas aeruginosa* ATCC 27853, *Proteus mirabilis* ATCC 25933, *Staphylococcus aureus* ATCC 25923, and *Enterococcus faecalis* ATCC 29212 were each suspended in 500 µL of 0.85% NaCl. Subsequently, each bacterial suspension was combined with 500 µL of fecal samples, resulting in a final volume of 1 mL. Then, samples were subjected to DNA extraction using the boiling method and subsequently amplified by nPCR.

### 2.8. Limit of Detection of nPCR at Various Incubation Times of Fecal Samples

Three milligrams of ESBL-negative fecal samples were suspended in 150 µL of 0.85% NaCl. *E. coli* samples harboring the *bla*_CTX-M_ gene were serially diluted two-fold in 0.85% NaCl to achieve a final bacterial concentration ranging from 10^8^ to 10^0^ CFU/mL. Eight sample batches were prepared, each consisting of 150 µL of each diluted bacteria, 150 µL of fecal sample, and 200 µL of peptone water. These batches were incubated at 0, 1, 2, 3, 4, 6, and 8 h, with incorporated fecal controls. DNA extraction was carried out using the boiling method and subjected to nPCR. The primary and nPCR LOD were compared at each time point.

### 2.9. Validation of nPCR for the Direct Detection of the bla_CTX-M_ Gene from Pig Feces

The mPCR, sPCR, and nPCR results from 136 fecal samples were validated against the PCR sequencing outcomes of ESBL-producing-*E. coli* isolates within the identical specimens. The sensitivity and specificity metrics were calculated using MedCalc software version 23.0.2 (https://www.medcalc.org/) accessed on 8 September 2024. The presence of ESBL genes between antibiotic-using and nonantibiotic-using farms, and among each farm, were analyzed using the Mann–Whitney *U* test with SPSS 29.0.1. A *p*-value < 0.05 was considered statistically significant.

## 3. Results

### 3.1. Antimicrobial Susceptibility Pattern and ESBL Detection

**Figure 1:**
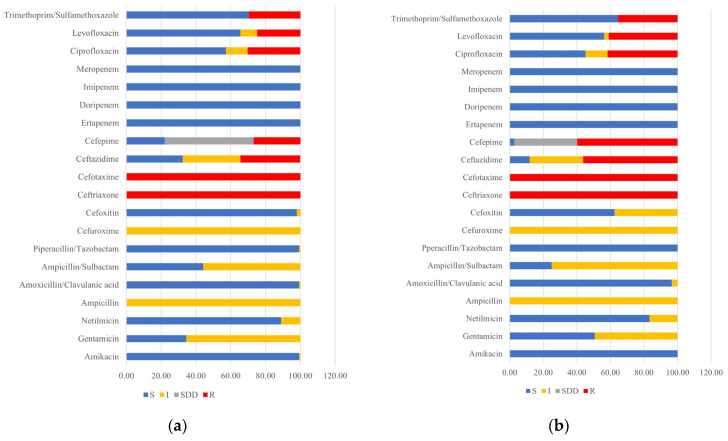
Percentage antimicrobial susceptibility profiles for (**a**) *E. coli* isolated from antibiotic-using farms and (**b**) *E. coli* isolated from nonantibiotic-using farms (S: susceptible, I: intermediate, SDD: susceptible-dose dependent, and R: resistant).

All 145 and 144 ESBL-producing *E. coli* isolated from antibiotic- and nonantibiotic-using farms demonstrated 100% non-susceptibility to ampicillin, cefuroxime, ceftriaxone, and cefotaxime. Surprisingly, the nonantibiotic-using farms had higher non-susceptible frequencies than antibiotic-using farms for other β-lactam and fluoroquinolone antibiotics including ampicillin/sulbactam, cefoxitin, ceftazidime, cefepime, ciprofloxacin, and levofloxacin (Figure 1a,b). Notably, no resistance to carbapenems was detected in either antibiotic- or nonantibiotic-using farms.

**Figure 2:**
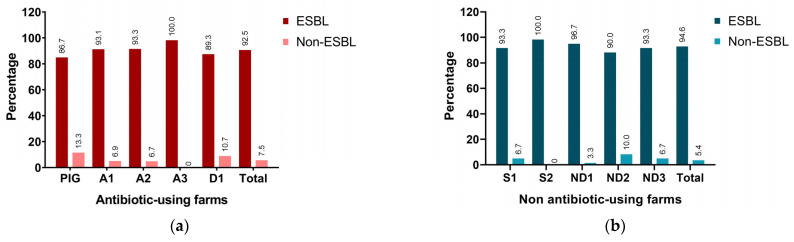
Phenotypic detection of ESBL-producing-*E. coli* isolates in (**a**) antibiotic-using and (**b**) nonantibiotic-using pig farms.

Of the 147 fecal samples in five antibiotic-using farms, 92.5% (136/147) were ESBL positive, divided into 86.7% (26/30), 93.1% (27/29), 93.3% (28/30), 100% (30/30), and 89.3% (25/28) from PIG, A1, A2, A3, and D1 farms, respectively, (Figure 2a). Among the 149 fecal samples in five nonantibiotic-using farms, 94.6% (141/149) were ESBL positive, divided into 93.3% (28/30), 100% (29/29), 96.7% (29/30), 90% (27/30), and 93.3% (28/30) from S1, S2, ND1, ND2, and ND3 farms, respectively, (Figure 2b). Moreover, at least 40% of ESBL-producing-*E. coli* isolates in PIG, A1, S1, and S2 farms were multi-drug-resistant strains (Figure S1). Phenotypic screening did not reveal a significant difference in ESBLs between antibiotic- and nonantibiotic-using farms (*p*-value > 0.05).

### 3.2. Characterization of ESBL Variants

A total of 289 isolates with an ESBL phenotype from antibiotic- (*n* = 145) and nonantibiotic-using (*n* = 144) farms were genotypically characterized for four common ESBL genes, including *bla*_CTX-M_, *bla*_TEM_, *bla*_OXA_, and *bla*_SHV_, using PCR sequencing. As is shown in Table 2, the frequency of ESBLs in antibiotic-using farms was 98.6% for *bla*_CTX-M_ and 53.1% for *bla*_TEM_, whereas nonantibiotic-using farms had frequencies of 99.3% for *bla*_CTX-M_ and 75% for *bla*_TEM_. All arise from either the CTX-M variant alone or combined with the TEM variants. The CTX-M-55 gene was the most common, with 85.5% and 95.1% in antibiotic- and nonantibiotic-using farms, respectively. A subtype of CTX-M-14 was greater in antibiotic- (13.1%) than in nonantibiotic-using farms (1.4%). Meanwhile, a few CTX-M-15 genes presented only in one nonantibiotic-using farms. TEM-1b, a non-ESBL variant, was dispersed among all of the farms. Only two and one ESBL isolates from the antibiotic- and nonantibiotic- using farms, respectively, did not carry those common ESBL genes. No significant difference in ESBL variants was revealed among each farm (*p*-value > 0.05) and between antibiotic- and nonantibiotic-using farms (*p*-value > 0.05).

### 3.3. Specificity of nPCR for the Direct Fecal Detection of bla_CTX-M_

**Figure 3:**
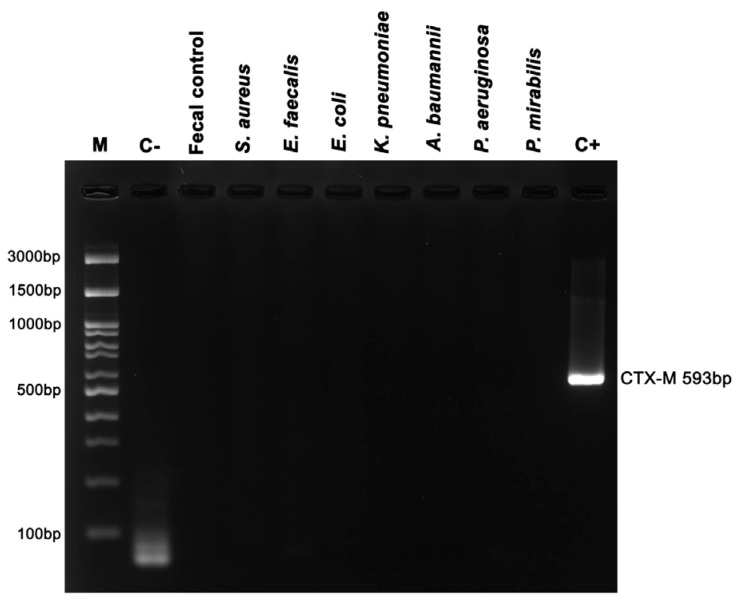
Specificity of nPCR for direct *bla*_CTX-M_ detection. Lane M: 100 bp DNA marker (Bio-helix, Taiwan), Lane C−: negative control, Lane 3: Fecal control, Lanes 4–10: ATCC bacterial strain controls, Lane C+: positive control.

No cross-reaction was observed with the reference bacterial strains, including *S. aureus* ATCC 25923, *E. faecalis* ATCC 29212, *E. coli* ATCC 25922, *K. pneumoniae* ATCC 700603, *A. baumannii* ATCC 19606, *P. aeruginosa* ATCC 27853, and *P. mirabilis* ATCC 25933 (Figure 3).

### 3.4. Limit of Detection of nPCR for the Direct Fecal Detection of bla_CTX-M_

The fecal samples were spiked with *E. coli* harboring the *bla*_CTX-M_ gene at concentrations ranging from 10^8^ to 10^0^ CFU/mL. To enhance the detection limit, all samples underwent enrichment in peptone and were cultivated from 0 to 8 h. The LODs of sPCR at 0–8 h ranged from 10^7^ to 10^2^ CFU/mL (Table 3 and Figure S2). All negative results in sPCR were subsequently examined using nPCR. The LOD of sPCR decreased when nPCR and a longer incubation time were applied as follows: from 10^7^ to 10^5^ CFU/mL at 0 h, 10^5^ to 10^4^ CFU/mL at 1 h, 10^4^ to 10^2^ CFU/mL at 2 h and 3 h, 10^3^ to 10^1^ CFU/mL at 4 h and 6 h, and 10^2^ to 1 CFU/mL at 8 h.

### 3.5. Comparison of the Direct Detection of the bla_CTX-M_ Gene from Pig Feces Using mPCR, sPCR, and nPCR Techniques

All 136 pig fecal samples were screened for four common ESBL genes by mPCR. The results showed the presence of *bla*_CTX-M_ in 30.2% (41/136) of samples, *bla*_TEM_ in 82.4% (112/136), *bla*_OXA_ in 11% (15/136), and *uspA* in 81.6% (111/136). However, the sPCR of *E. coli* isolates from the same fecal samples revealed 100% of the *bla*_CTX-M_ gene. Meanwhile, sPCR and nPCR results from direct fecal detection showed 69.9% and 91.9% positivity for the *bla*_CTX-M_ gene. The sensitivity for the direct detection of the *bla*_CTX-M_ gene using the mPCR, sPCR, and nPCR techniques was 30.15%, 69.85%, and 91.91%, respectively (Table 4).

## 4. Discussion

The acceleration of livestock’s antimicrobial resistance (AMR) genes substantially impacts upon the global economy and food security. The surveillance and control of antimicrobial resistance (AMR) genes are a great priority in the One Health approach [15]. Pigs are one of the reservoirs for AMR, especially ESBL-producing bacteria. The overuse or misuse of antibiotics imposes selective pressure, leading to a steady increase in antibiotic resistance in livestock systems, which is transferred to humans and the environment. However, the incidence of AMR has been noticed in animals without antibiotic usage [5]. Thus, antibiotic use may not be the only factor influencing the stability of AMR genes in farms.

ESBL genes are commonly located on plasmids within transposons or insertion sequences. This arrangement facilitates horizontal gene transfer, enabling related bacteria or even bacteria from different families to acquire antibiotic-resistant genes. The acquisition of these genetic elements can lead to the limited effectiveness of therapy, hindering the ability to combat infections effectively [16]. In our study, the high prevalence of ESBL-producing *E. coli* with MDR profiles existed in antibiotic- and nonantibiotic-using pig farms, with no significant differences. The CTX-M-55 gene, which is usually found in commensal *E. coli*, was predominantly found in the *E. coli* isolated in our study [17,18,19]. A few discordant results of ESBL-producing isolates that did not carry the four common ESBL genes in this study might be due to other uncommon ESBL variants or antibiotic-resistant mechanisms [20]. The CTX-M-55 gene was most detected in pork samples from Bangkok retail markets and pig cecum samples in Thailand [16]. CTX-M-14 and CTX-M-15, clinically associated variants, were observed in low frequencies in antibiotic- and nonantibiotic-using farms. Similarly, clinical CTX-M variants were recently found in pig farms [3]. In Mecklenburg–Western Pomerania, comparable ESBLs were found on conventional and organic pig farms, with CTX-M variants being more common [21]. Although low or no use of antibiotics in pig farms reduces AMR in *E. coli*, a basal level of MDR *E. coli* persists on farms due to the transmission and recycling of specific clones within distinct pig groups [22]. The presence of ESBL strains in nonantibiotic-using farms might be due to the farm environment, clonal expansion, and horizontal gene transfer resulting in a shift in phylogenetic groups [23]. The abundance and diversity of ESBL genes may not depend on antibiotic use, and high levels of specific genes are sustainable throughout microbial populations in pig farms. AMR originating in pig farms is widely disseminated in the production cycle. Moreover, AMR from human carriers are the external transmission sources that make the AMR gene dynamics with the clinical strains [24].

Although genotypic detection from isolated colonies yields higher sensitivity and lower detection limits, the limitations lie in the cumbersome process and long incubation time of conventional culture, bacterial identification, and antimicrobial susceptibility testing. Biosafety risks are another issue when dealing with pathogenic organisms contained in specimens. The stool is a problematic specimen for DNA extraction due to different substances derived from foods, cell residues, and host DNA contamination. Moreover, the presence of interferences inhibits amplification, leading to low sensitivity. Fecal specimens present challenges for direct bacterial DNA extraction, which affects their usefulness in detecting fecal pathogens and studying the gut microbiome.

The isolation and purification of DNA from fecal samples are crucial to ensure a high yield and quality of isolated nucleic acids. Especially in gut microbiome studies, the purity of the extracted DNA has substantial effects on downstream library preparation, microbial diversity, and taxonomic profiling [25]. The traditional boiling method is simple and low cost, but the purity and yields are problematic. However, sufficient DNA for successful downstream PCR or other amplification principles has been observed in many studies [26,27,28]. The direct boiling of fecal samples across a wide range of concentrations for gut microbiome studies revealed a similar pattern of bacterial communities and highly consistent frequencies of operational taxonomic units compared to those obtained from most commercial kits [29]. In addition, DNA preparation by boiling is suitable for identifying amplified targets from a large quantity of samples in a limited time [30]. The simple boiling method was compared with five commercial kits, including the QIAamp Stool Mini kit (Qiagen, Germantown, MD, USA), the Bioeasy Fecal Magnetic Isolation Kit, the Bioeasy Animal Column Isolation kit, the TIANamp Stool DNA kit, and the UltaraClean Fecal DNA kit, for subsequent use in fecal microbiome analysis. All methods resulted in genomic DNA degradation, although only Qiagen and TIANamp yielded visible amounts of genomic DNA on gel electrophoresis; PCR amplification results from all extraction methods were not different [29].

Although the mPCR is beneficial for detecting multiple targets, amplification from DNA extracted from stool samples yielded very low sensitivity. The low DNA purity largely influences mPCR rather than sPCR and nPCR reactions. The disadvantages of mPCR are the lack of detection sensitivity for a single target, high amount of initial DNA templates, primer competition, and non-specific amplification products [31]. The nPCR re-amplifies the low abundance of the first PCR products, enhancing sensitivity and specificity [32]. However, a high contamination risk is a major disadvantage due to possible carry-over contamination of primary PCR products. Substantial caution must be exercised by separating PCR areas, using the uracil-N-glycosylase (UNG) system, and cleaning the space, pipettes, and other equipment after each round of PCR. The more prolonged incubation of stool in an enrichment medium also increases the bacterial number to a sufficient LOD. Enrichment and dilution of natural PCR inhibitors in stool samples enhance sensitivity for *Shigella* spp. and enteroinvasive *E. coli* detection [33]. Directly detecting pathogens or antimicrobial-resistance genes in specimens by nPCR is advantageous in cases of low favorable culture rates and a tiny number of initial microorganisms.

## 5. Conclusions

The antimicrobial susceptibility profiles and the presence of ESBL genes did not differ between antibiotic- and nonantibiotic-using farms. CTX-M-55 predominated in commensal *E. coli* isolated from pigs. Direct detection of ESBL genes from pig fecal samples using nPCR with a longer incubation time in an enrichment medium enhanced the sensitivity. Whole-genome analysis of *E. coli* isolates from antibiotic- and nonantibiotic-using farms will be studied further to deeply compare all data, especially resistomes, virulence genes, pathotypes, the phylogenetic relationship, and mobile genetic elements, which will be beneficial for the assessment of risk factors and antimicrobial gene persistence on pig farms. Comparison of phenotypic and genotypic antimicrobial-resistant profiles of *E. coli* isolated from diseased pigs and commensal strains also represents our future challenge.

## Figures and Tables

**Table 1:** Primer sequence and amplicon sizes.

Target Genes	Primer Sequence (5′-3′)	Amplicon Size (bp)	References
*bla*_CTX-M_	F-ATGTGCAGYACCAGTAARGTKATGGC	593	[13]
	R-TGGGTRAARTARGTSACCAGAAYCAGCGG		
*bla*_TEM_	F-AGTGCTGCCATAACCATGAGTG	431	[14]
	R-CTGACTCCCC GTCGTGTAGATA		
*bla*_OXA_	F-ATTATCTACAGCAGCGCCAGTG	296	
	R-TGCATCCACGTCTTTGGTG		
*bla*_SHV_	F-GATGAACGCTTTCCCATGATG	214	
	R-CGCTGTTATCGCTCATGGTAA		
*uspA*	F-AATGCAGGCTACCCAATCAC	162	[3]
	R-GGTGTTGATCAGCTGACGTG		

**Table 2:** Percentage detection of CTX-M and TEM variant genes in antibiotic- and nonantibiotic-using farms. Statistically significant differences are shown according to the degree of significance.

ESBL Variants	Antibiotic-Using Farms							Nonantibiotic-Using Farms						
	No (%)							No (%)						
	PIG (*n* = 28)	A1 (*n* = 30)	A2 (*n* = 29)	A3 (*n* = 32)	D1 (*n* = 26)	Total (*n* = 145)	*p*-Value *	S1 (*n* = 28)	S2 (*n* = 29)	ND1 (n = 32)	ND2 (*n* = 27)	ND3 (*n* = 28)	Total (*n* = 144)	*p*-Value *
CTX-M-55, TEM-1b	12 (42.9)	9 (30)	-	20 (62.5)	11 (42.3)	52 (35.9)	0.406	28 (100)	29 (100)	12 (37.5)	12 (44.4)	27 (96.4)	108 (75)	0.406
CTX-M-55	13 (46.4)	6 (20)	20 (69)	10 (31.3)	15 (57.7)	64 (44.1)	0.406	-	-	19 (59.4)	9 (33.3)	-	28 (19.4)	0.406
CTX-M-55, TEM-176	-	-	8 (27.6)	-	-	8 (5.5)	0.406	-	-	-	-	-	-	1.000
CTX-M-55, CTX-M-14	-	-	-	-	-	-	1.000	-	-	-	-	1 (3.6)	1 (0.7)	0.406
CTX-M-14, TEM-1b	1 (3.6)	11 (36.7)	1 (3.4)	2 (6.3)	-	15 (10.3)	0.406	-	-	-	-	-	-	1.000
CTX-M-14	2 (7.1)	2 (6.7)	-	-	-	4 (2.8)	0.406	-	-	1 (3.1)	1 (3.7)	-	2 (1.4)	0.406
CTX-M-15	-	-	-	-	-	-	1.000	-	-	-	5 (18.5)	-	5 (3.5)	0.406
TEM-1b	-	2 (6.7)	-	-	-	2 (1.4)	0.406	-	-	-	-	-	-	1.000

* Mann–Whitney U test with significance at *p*-value < 0.05.

**Table 3:** LOD of sPCR and nPCR for direct detection of *bla*_CTX-M_ in pig feces from 0 to 8 h.

CFU/mL	Methods	Incubation Time						
		0 h	1 h	2 h	3 h	4 h	6 h	8 h
10^8^	sPCR	+	+	+	+	+	+	+
	nPCR	+	+	+	+	+	+	+
10^7^	sPCR	+w	+	+	+	+	+	+
	nPCR	+	+	+	+	+	+	+
10^6^	sPCR	−	+	+	+	+	+	+
	nPCR	+	+	+	+	+	+	+
10^5^	sPCR	−	+w	+	+	+	+	+
	nPCR	+w	+	+	+	+	+	+
10^4^	sPCR	−	−	+w	+w	+	+	+
	nPCR	−	+w	+	+	+	+	+
10^3^	sPCR	−	−	−	−	+w	+	+
	nPCR	−	−	+w	+	+	+	+
10^2^	sPCR	−	−	−	−	−	+w	+w
	nPCR	−	−	+w	+w	+	+	+
10^1^	sPCR	−	−	−	−	−	−	−
	nPCR	−	−	−	−	+w	+w	+
10^0^	sPCR	−	−	−	−	−	−	−
	nPCR	−	−	−	−	−	−	+w

Plus (+) sign represents the presence of the *bla*_CTX-M_ amplicon; the minus (−) sign represents the absence of the *bla*_CTX-M_ amplicon; the weak plus (+w) sign represents the presence of the faint *bla*_CTX-M_ amplicon.

**Table 4:** Comparison among mPCR, sPCR, and nPCR for direct detection of the *bla*_CTX-M_ gene from pig fecal samples.

PCR from *E. coli* Isolates	Direct Detection from Pig Fecal Samples								
	mPCR			sPCR			nPCR		
	+	−	Total	+	−	Total	+	−	Total
*bla*_CTX-M_ +	41	0	41	95	0	95	125	0	125
*bla*_CTX-M_ −	95	0	95	41	0	41	11	0	11
Total	136	0	136	136	0	136	136	0	136
	Sensitivity: 30.15% CI 95% [22.58–38.60%] PPV: 100% CI 95% [91.40–100.00%]			Sensitivity: 69.85% CI 95% [61.40–77.42%] PPV: 100% CI 95% [96.19–100.00%]			Sensitivity: 91.91% CI 95% [85.99–95.89%] PPV: 100% CI 95% [97.09–100.00%]		

## Data Availability

The datasets generated during the current study are available in the GenBank NCBI repository, with the accession numbers OR672090–OR672093 and OR680712.

## Supplementary Materials

The following supporting information can be downloaded at: https://www.mdpi.com/article/10.3390/ani14182630/s1, Figure S1: The antimicrobial susceptibility profiles of ESBL-producing *E. coli* isolated from 10 farms; Figure S2: LOD of sPCR and nPCR on agarose gel for direct *bla*_CTX-M_ detection.
