# Supplementary figures and images for: Antimicrobial Susceptibility of Commensal Escherichia coli from Pig Fecal Samples and Enhanced Sensitivity for Direct Detection of the blaCTX-M Gene by Nested PCR
Source: Animals (Basel). 2024 Sep 10;14(18):2630. doi: 10.3390/ani14182630 (PMC11428893; doi:10.3390/ani14182630)

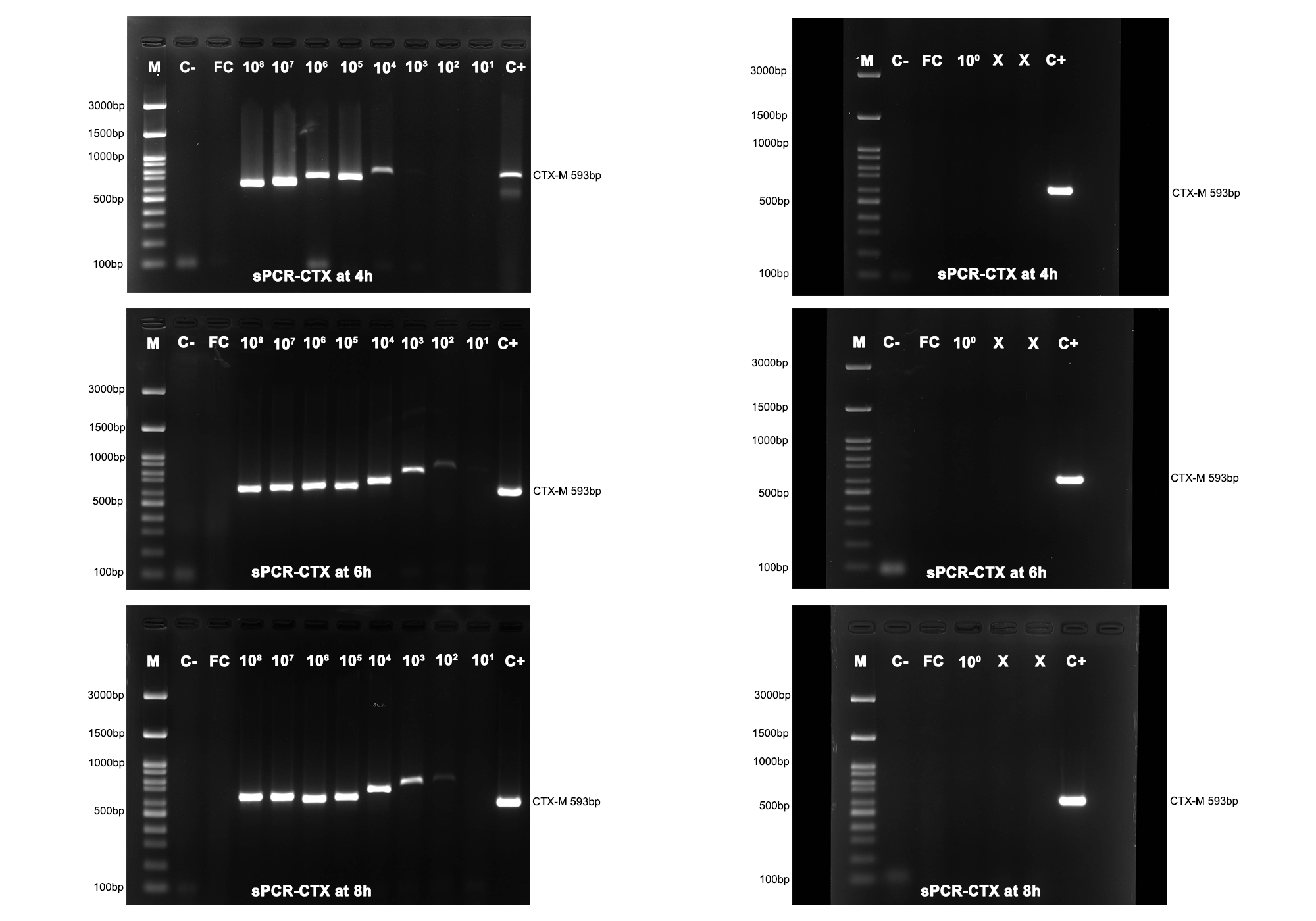

Supplement: Supplementary file 1 [file animals-14-02630-s001.zip › Figure S2. LOD of sPCR (at 4-8h).tif]

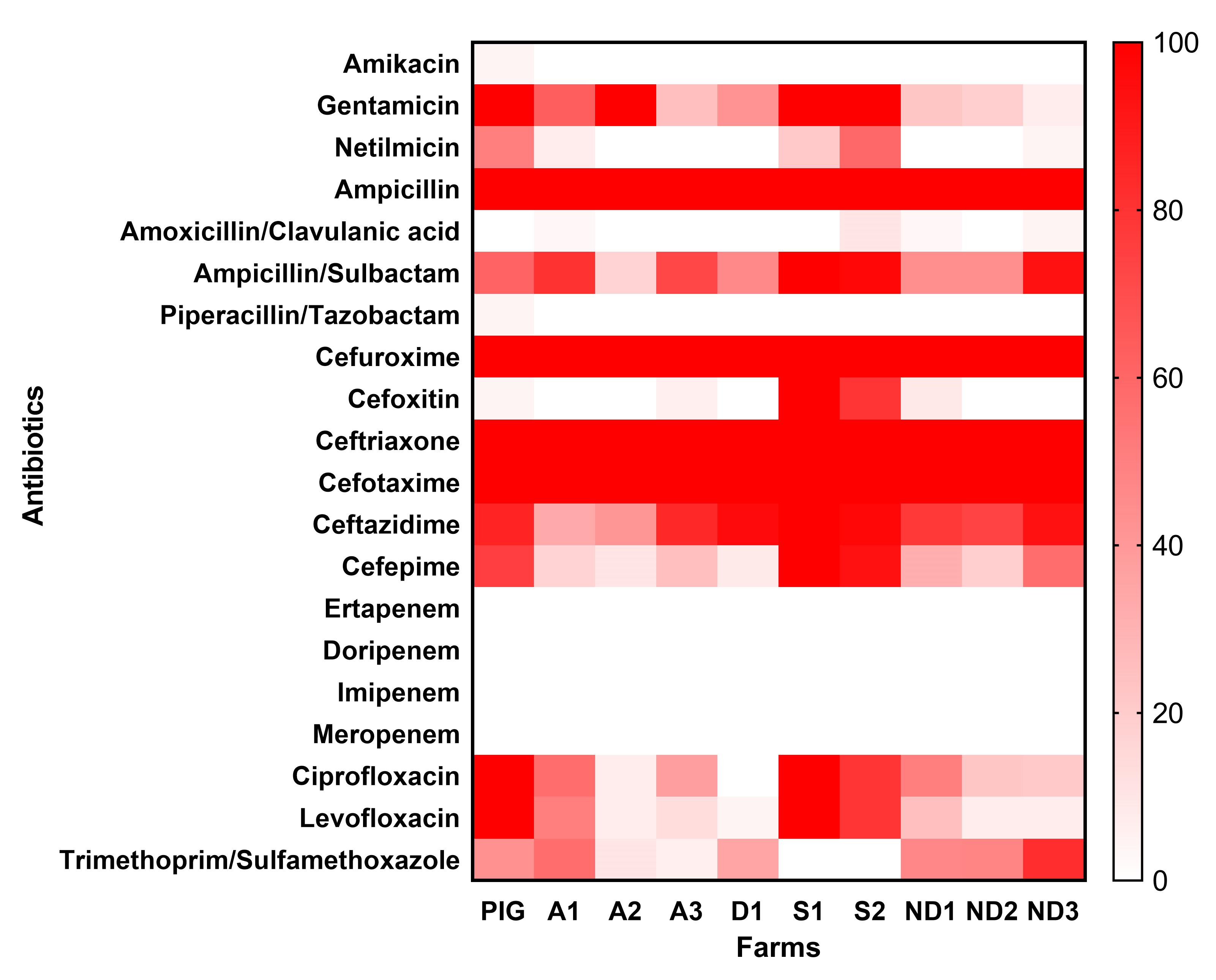

Supplement: Supplementary file 1 [file animals-14-02630-s001.zip › Figure S1.tiff]

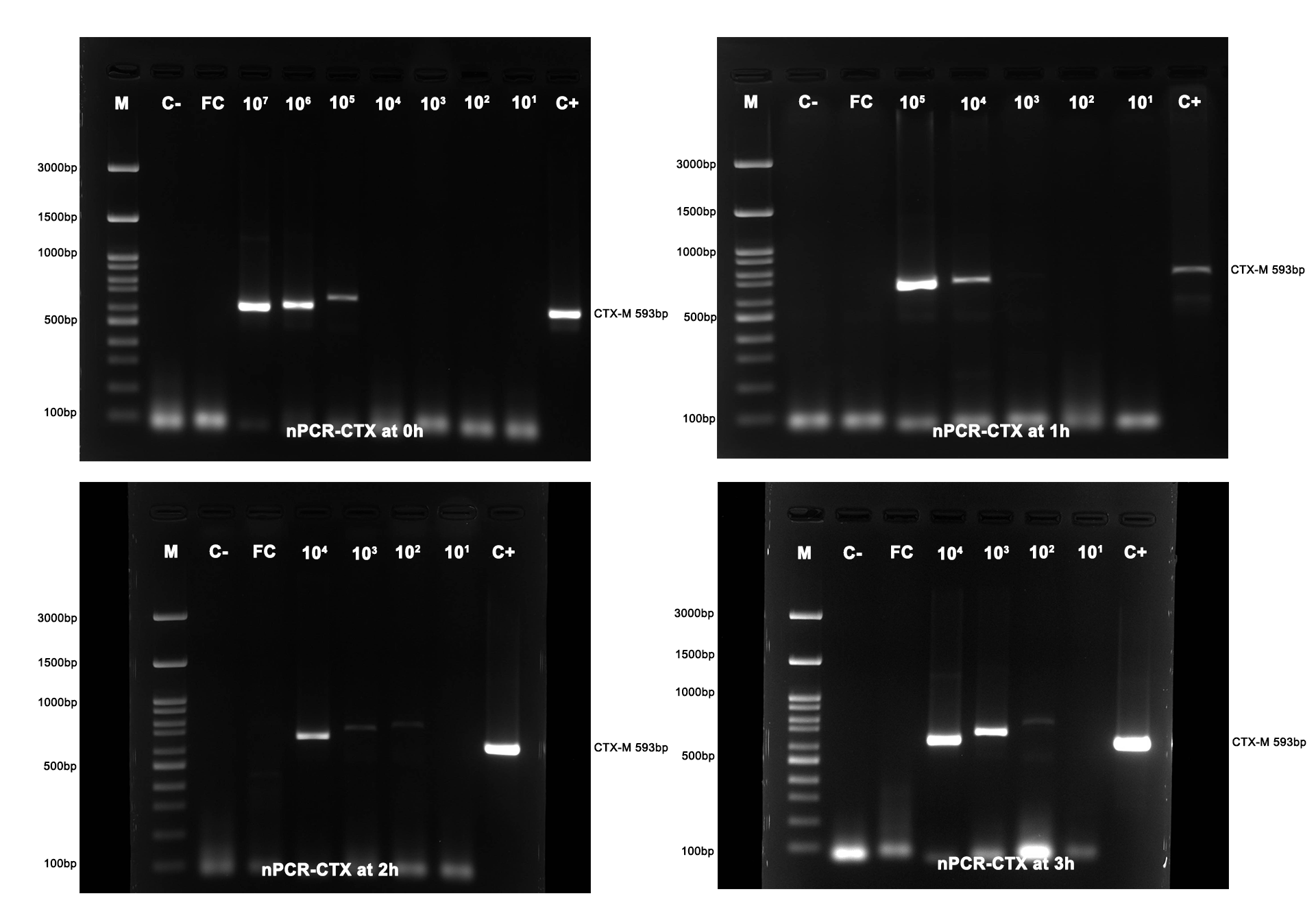

Supplement: Supplementary file 1 [file animals-14-02630-s001.zip › Figure S2. LOD of nPCR (at 0-3h).tif]

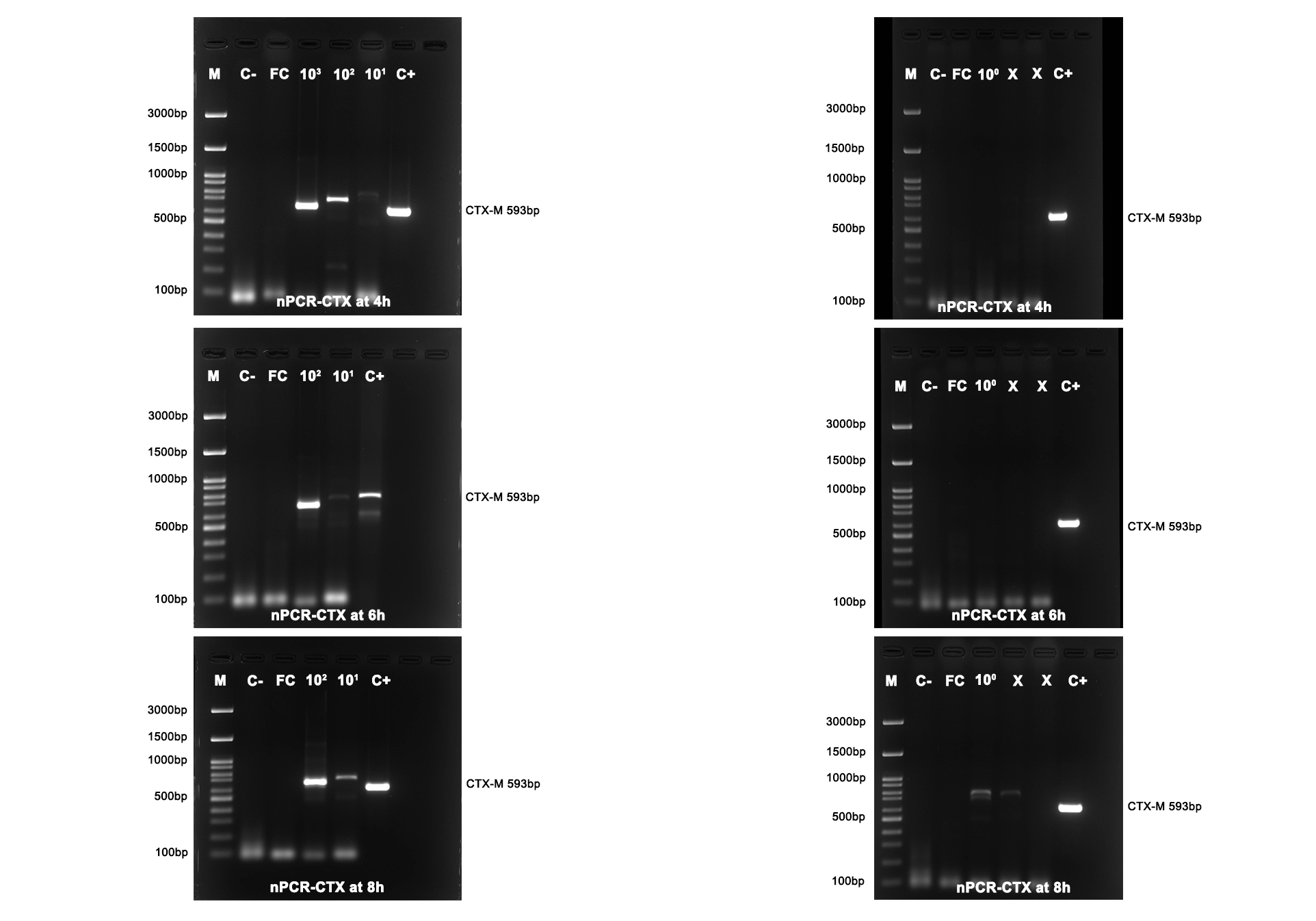

Supplement: Supplementary file 1 [file animals-14-02630-s001.zip › Figure S2. LOD of nPCR (at 4-8h).tif]

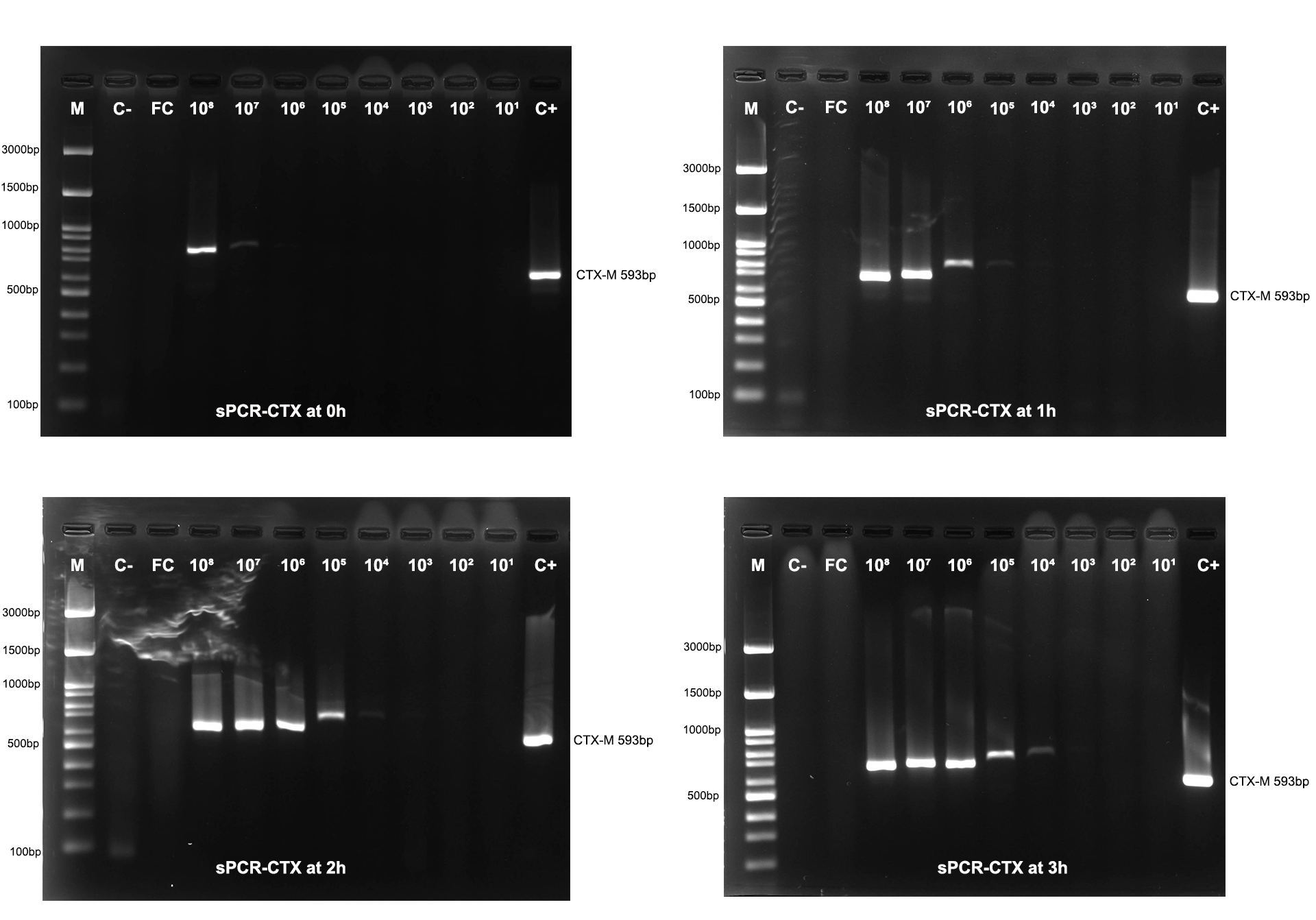

Supplement: Supplementary file 1 [file animals-14-02630-s001.zip › Figure S2. LOD of sPCR (at 0-3h).tif]
